# Antimicrobial Denture Material Synthesized from Poly(methyl methacrylate) Enriched with Cannabidiol Isolates

**DOI:** 10.3390/molecules30040943

**Published:** 2025-02-18

**Authors:** Kazi Tahsin, William Xu, David Watson, Amin Rizkalla, Paul Charpentier

**Affiliations:** 1 Biomedical Engineering, Western University, London, ON N6A 3K7, Canada; arizkall@uwo.ca; 2 Chemical & Biochemical Engineering, Western University, London, ON N6A 5B9, Canada; zxu27@uwo.ca; 3 Microbiology & Immunology, Western University, London, ON N6A 5C1, Canada; dwatso25@uwo.ca

**Keywords:** denture material, antimicrobial coating, cannabidiol, poly(methyl methacrylate), UV curing

## Abstract

Cannabidiol (CBD), derived from the Cannabis plant, has shown potential in dentistry for its antimicrobial properties, particularly against oral bacteria. Denture-associated infections, a common issue among denture wearers, present a challenge in antimicrobial enhancements to poly(methyl methacrylate) (PMMA), the primary material for dentures due to its favorable physical and aesthetic qualities. To address this, researchers developed PMMA denture coatings infused with CBD nanoparticles. The CBD coatings were synthesized using UV curing and characterized via ^1^H NMR, SEM, and FTIR spectroscopies. Antimicrobial activity was assessed against *Staphylococcus aureus*, *Escherichia coli*, and *Streptococcus agalactiae*. CBD demonstrated significant bactericidal effects on Gram-positive bacteria with a minimum inhibitory concentration (MIC) of 2–2.5 µg/mL and a minimum bactericidal concentration (MBC) of 10–20 µg/mL but was ineffective against planktonic Gram-negative bacteria. However, biofilm studies revealed a 99% reduction in biofilm growth for both Gram-positive and Gram-negative bacteria on CBD-infused PMMA compared to standard PMMA. The CBD disrupted bacterial cell walls, causing lysis. Dissolution studies indicated effective release of CBD molecules, crucial for antimicrobial efficacy. This study highlights CBD’s potential for antibiotic-free denture coatings, reducing dental biofilms and plaque formation, and improving oral health outcomes.

## 1. Introduction

Poly(methyl methacrylate), or PMMA, is frequently employed as a biomaterial in the construction of both partial and complete dentures. This choice is driven by the material’s advantageous physical and mechanical properties, its ease of fabrication, and its cost-effectiveness [1]. However, PMMA is constrained by its absence of antimicrobial properties causing certain limitations. Consequently, there is a rising interest in enhancing PMMA integrated with antimicrobial characteristics. Methods involve integrating antimicrobial agents like nanoparticles or quaternary ammonium compounds into the PMMA matrix [2]. Other approaches involve altering the PMMA surface using antimicrobial agents coated to the surface or grafting techniques. These alterations are designed to inhibit the growth and colonization of bacteria and fungi on the denture surface, thereby mitigating the risk of oral infections and other associated health complications [3]. In the oral cavity, primary colonizers of hard/enamel surfaces include mostly Gram-positive *Streptococcus* spp. [4], *Staphylococcus aureus*, commonly known as *MRSA* [5], *Escherichia coli* [6,7], and other species including elevated levels of yeasts (primarily *Candida* spp.) [5,7].

Traditional antimicrobial agents may work against a broad spectrum of pathogens but may have significant side effects (e.g., diarrhea, colitis, shortage of commensal bacteria). Non-antibiotic approaches not only bring positive results in pathogen prevention but also reduce the potential of bacterial resistance [8]. As numerous countries are beginning to enforce stricter regulations on antibiotics, the need to adopt alternative agents on a commercial scale is becoming important.

Plants and their extracts have natural antibacterial properties and their ability to control oral pathogens has only recently been scientifically confirmed; additionally, they are recognized as promising against antibiotic-resistant bacteria [8]. Plants have evolved a variety of intricate mechanisms to counter continuous microbial threats in their environment. Since plants lack cell-based immune responses, they have had to devise alternative strategies for eliminating microorganisms. Plants include various useful components, including polyphenols, phenols, micronutrients, phytochemicals, and essential oils.

*Cannabis sativa* L., known as the cannabis plant, produces over 120 different compounds which have relatively unknown pharmacological profiles. The constituents of C. Sativa consist of the psychoactive tetrahydrocannabinol (THC), non-psychoactive cannabidiol (CBD), mildly psychoactive cannabinol (CBN), followed by the parent molecule cannabigerol (CBG), cannabichrom (CBC), and, in low amounts, psychoactive cannabidiol (CBND) [9,10]. CBD is a non-psychotropic compound and is biocompatible [11]. The antimicrobial characteristics of CBD have been used against multi-resistant bacterial infections including *S. aureus*, *S. epidermidis*, and other Gram-negative bacteria [10]. The antimicrobial mechanism includes having a resorcinol moiety and a long pentyl tail for disrupting bacterial cytoplasmic cell walls [10]. CBD provides the potential for various applications in the fields of dentistry and oral medicine. CBD has been used for oral care (e.g., toothpaste, mouthwash, dental floss), tooth whitening ability, injury repair potential, antifungal and anti-inflammatory agents, analgesic effects, and even treatment of viral infections [12,13,14,15,16,17,18,19,20,21,22,23,24]. CBD has been found to improve the recovery process of ulcers by lowering the number of colony counts of bacterial strains in dental plaque when compared to well-established chemical oral care solutions [25]. CBD has antibacterial capabilities which may be beneficial in lowering the colony count of bacterial strains in dental plaque, thereby controlling and reducing bacterial-induced inflammatory periodontal infections [25,26].

In this research, we have synthesized an antibiotic-free polymer coating enriched with CBD for coating the surfaces of denture materials. The monomer, methyl methacrylate (MMA), was synthesized under UV irradiation with the addition of CBD, and the necessary UV curing duration was examined. The use of UV radiation in the polymerization of methyl methacrylate (MMA) to poly(methyl methacrylate) (PMMA) has benefits over traditional heat-initiated polymerization. UV curing provides much faster (15 min) polymerization over thermal polymerization which takes around 6 h to 10 h [27]. Heat curing also requires higher temperatures, 60 °C to 90 °C to achieve high yields, whereas UV curing can be performed in lower temperatures, reducing energy costs in achieving similar yields [27]. The objective of this research article was to synthesize a denture surface from PMMA loaded with CBD for bactericidal effects. After the spectroscopic, microscopic, and analytical characterization of the coating, its antibacterial and antibiofilm activities and drug-release profile were examined to show the utility of this new coating. The antimicrobial activity of the CBD was evaluated before synthesizing it into the PMMA material on *Staphylococcus aureus*, *Escherichia coli*, and *Streptococcus agalactiae*. Antibiofilm studies were conducted against *S. aureus*, *E. coli*, and *S. agalactiae* biofilms. The synthesized materials may have no significant effect on the biofilms of the strains, or they only affect some of the strains but not others.

## 2. Results and Discussion

### 2.1. Coating Characterization and Analysis

To assess the purity of the industrial CBD crystal samples, CBD fractions were analyzed and characterized using ^1^H NMR spectroscopy on a Varian Inova—600 (California, USA). The validation of the ^1^H NMR results involved the quantification of cannabinoids of interest by comparing the peak intensities and integration numbers of our sample to those documented in the literature [28]. The unique feature of CBD is that the proton signal of the aromatic protons is about 5.9–6.28 (Supplementary Materials, Figure S1) and clearly broadens in the ^1^H NMR spectrum in contrast to signals of the other cannabinoids [28].

Of interest for polymer synthesis is the available vinyl group on C8-C9 of the CBD structure. The copolymerization process involved the addition of an MMA monomer, which was polymerized in the presence of the extracted CBD through a UV curing method using the UV photo initiator HMP. HMP functions by absorbing UV light energy, generating free radicals that initiate polymerization. In this context, HMP absorbs UV light within the wavelength range of 220 to 390 nm, to initiate synthesis reactions.

**Figure 1:**
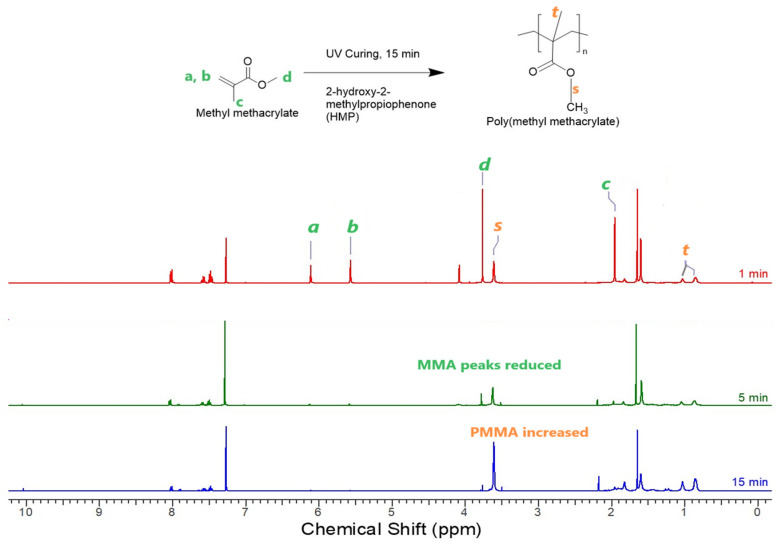
^1^H NMR spectra of PMMA coatings with 1, 5, and 15 min UV curing.

To determine the necessary UV curing time for polymerization completion, the system comprising MMA, CBD, PMMA, and HMP was explored. PMMA, acting as a thickener, was incorporated at 0.08 mol of MMA. The investigation involved examining the ^1^H NMR spectra of UV-cured PMMA coating samples with varying UV curing times, as depicted in Figure 1. The peaks of 0.80–1.2 ppm corresponded to the –CH_3_ group of PMMA (*t*) and other peaks of 6.25–5.58 corresponded to MMA peaks (*a*, *b*).

Analysis was conducted on the UV curing process to observe if the characteristic peaks of MMA were still evident at 3.76, 6.11, and 5.57 ppm, which would suggest an incomplete conversion of the MMA monomer. At 5 min, the MMA peaks of 6.11 ppm and 5.57 ppm were still visible, and the curing time was extended until all the MMA peaks disappeared at 15 min or beyond, indicating complete polymerization. The –OCH_3_ peaks of PMMA at 3.6 ppm were highest at 15 min in comparison to those at 1 and 5 min. The C=C bond peaks (*a*, *b*) at 6.11 and 5.57 ppm were seen in small amounts at 5 min, but disappeared at 15 min, suggesting complete conversion of MMA to PMMA. The amount of conversion is essential in maintaining the uniformity of the coating and unreacted monomer may affect the thickness and the quality of the layer with respect to uniformity.

### 2.2. Antimicrobial Studies

#### 2.2.1. MIC Studies

Minimum inhibitory concentration values allow us to obtain satisfactory ideas about the potency of CBD before use as a coating against different pathogenic microorganisms. The results of the in vitro effects of CBD dissolved in the solvent DMSO against pathogenic yeast and bacteria are demonstrated in Table 1. According to the obtained MIC results, >*S. aureus* > *E. coli*. DMSO is known for its antimicrobial properties and enhancing other compounds’ bioactivity, so DMSO alone was tested in the no CBD groups. CBD was proven to be ineffective against Gram-negative bacteria. This type of bacteria contains lipopolysaccharides and proteins with very few phospholipids on the outer leaflet of the outer membrane, which is a critical element in the membrane’s effective resistance. PMMA being a large macromolecule, when combined with hydrophobic CBD, the membrane of *E. coli* is not permeable to macromolecules and limits diffusion of hydrophobic compounds [29]. The synthesized coating suppressed the growth of *S. agalactiae* more effectively than other pathogens. Our research findings are consistent with the MIC values observed for *S. aureus* and *S. agalactiae* in [30], ranging from 1 to 5 µg/mL, which closely resemble our own results. Our results for MIC studies on Gram-negative bacteria were also consistent with previous reports [30], where CBD was inactive against 20 species of Gram-negative bacteria (*E. coli*, *Klebsiella pneumoniae*, *Pseudomonas aeruginosa*, and *Acinetobacter baumannii*) [26]. 

#### 2.2.2. Zone of Inhibition Studies

The antimicrobial activities of CBD were evaluated against each microbe using the disk diffusion method. The results in Table 1 show that the solvent DMSO had no significant effect on the antimicrobial activity of the purified CBD. The strongest inhibitory activity was observed by CBD against *S. agalactiae* and then *S. aureus*. The ZOI studies demonstrated CBD had no effect on Gram-negative *E. coli*, because of them having an outer membrane and more protection. This protective layer in Gram-negative bacteria is more resistant to antibiotics than Gram-positive bacteria. Most Gram-negative bacteria show innate resistance to many antimicrobial therapies due to the presence of lipopolysaccharides [31].

### 2.3. Bactericidal Studies

The observed MBC of CBD was determined by spot plating the bacterial suspensions from the MIC culture tubes on fresh agar plates for the three microorganisms. The lowest concentration resulting in no viable bacterial colonies was reported as MBC and recorded in Table 1. MBC represents the lowest concentration of an antimicrobial agent required for cell death.

**Figure 2:**
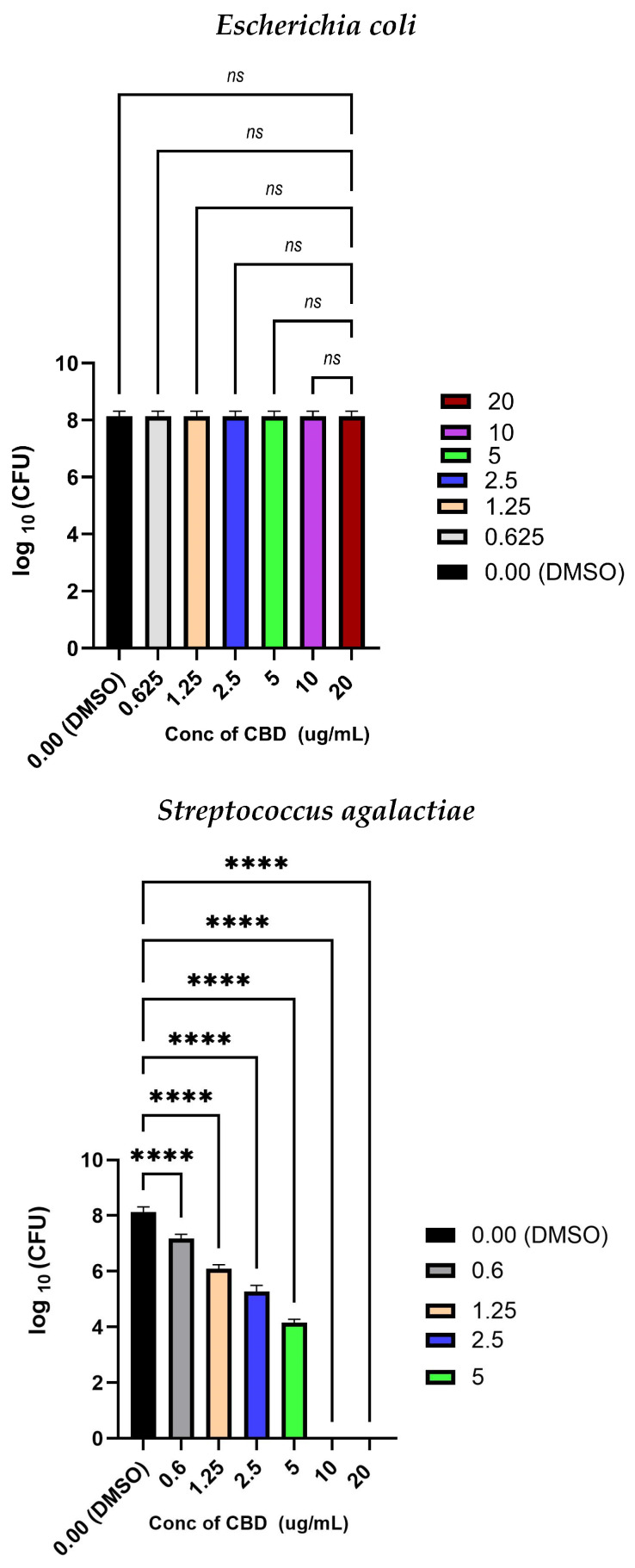

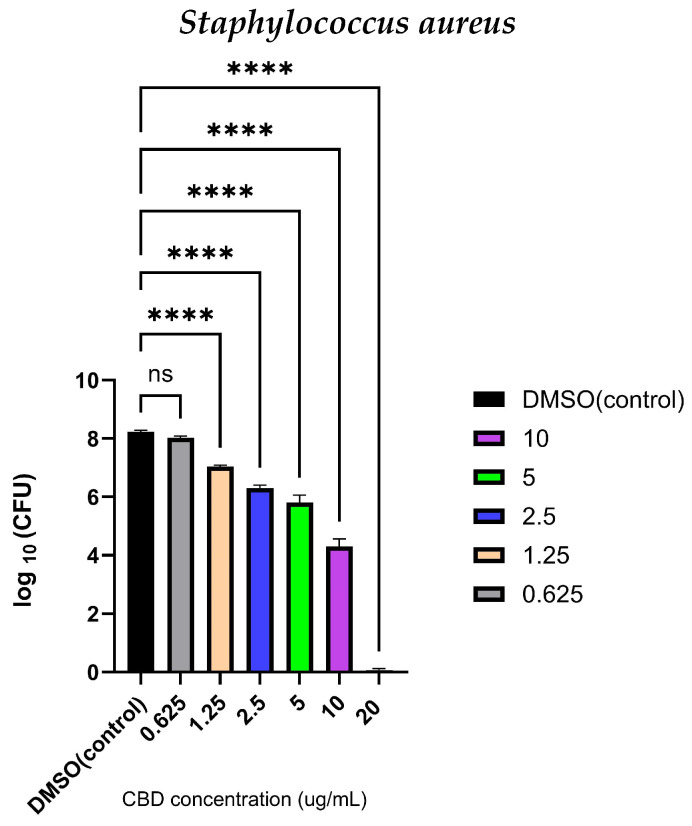
CBD concentration kill curve against *S. agalactiae*, *E. coli*, and S. *aureus*, with the significant difference denoted by *. Highly significant is denoted by ****, unsignificant is denoted by *ns*. Tested by ANOVA and Tukey’s multiple comparison.

A one-way ANOVA test along with Tukey’s multiple comparison tests were used to compare the coated MMA with no CBD and the coatings synthesized with different concentrations of CBD (Figure 2) for each pathogen. The *p*-values were noted for each pathogen. There was a significant difference between the values with CBD, and the ones treated with only solvent DMSO were evaluated for *S. aureus* and *S. agalactiae*. This showed that we can accept the hypothesis that the groups treated with CBD and the ones that had no CBD were statistically significantly different from each other and CBD concentration influences the log CFU counts of the cells for Gram-positive bacteria. According to the obtained MBC outcomes, CBD caused cell death of *S. agalactiae* more effectively than *S. aureus*. The bactericidal effects of CBD were *S. agalactiae* > *S. aureus* > *E. coli*.

### 2.4. Antibiofilm Studies

#### 2.4.1. Quantitative Analysis of Biofilms

**Figure 3:**
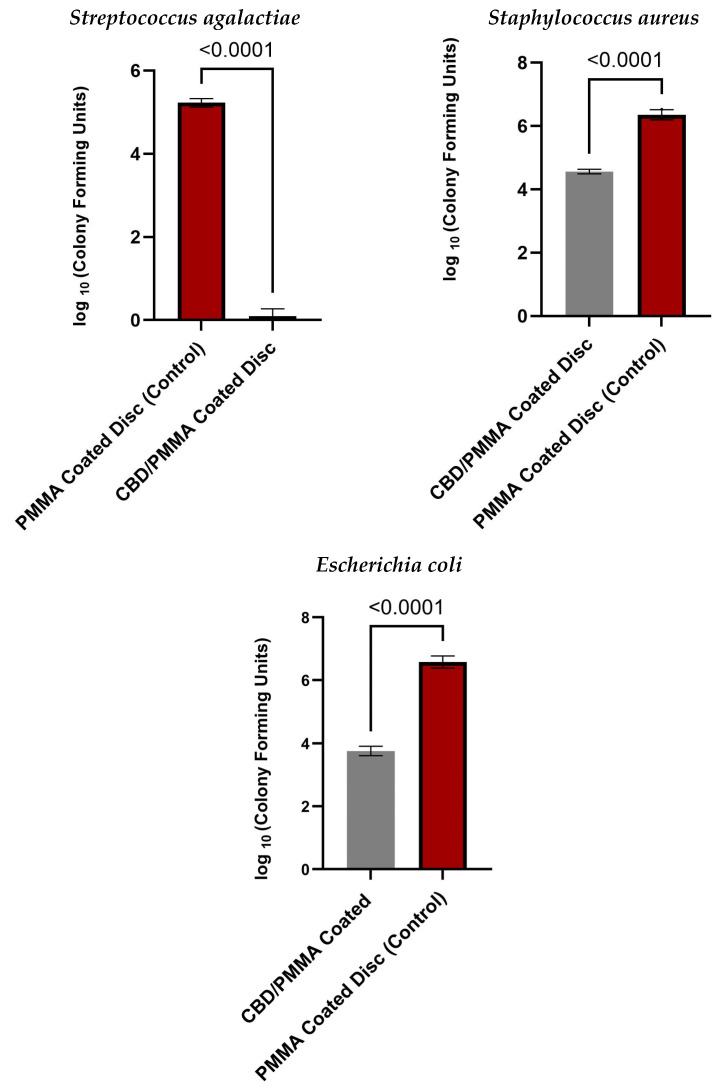
Log (CFU) counts of bacteria in MMA coatings and PMMA/CBD coatings after 24 h incubation with *S. agalactiae*, *S. aureus*, and *E. coli* biofilms. *p*-value of 0.001 resembles a high significant difference between the two groups.

**Figure 4:**
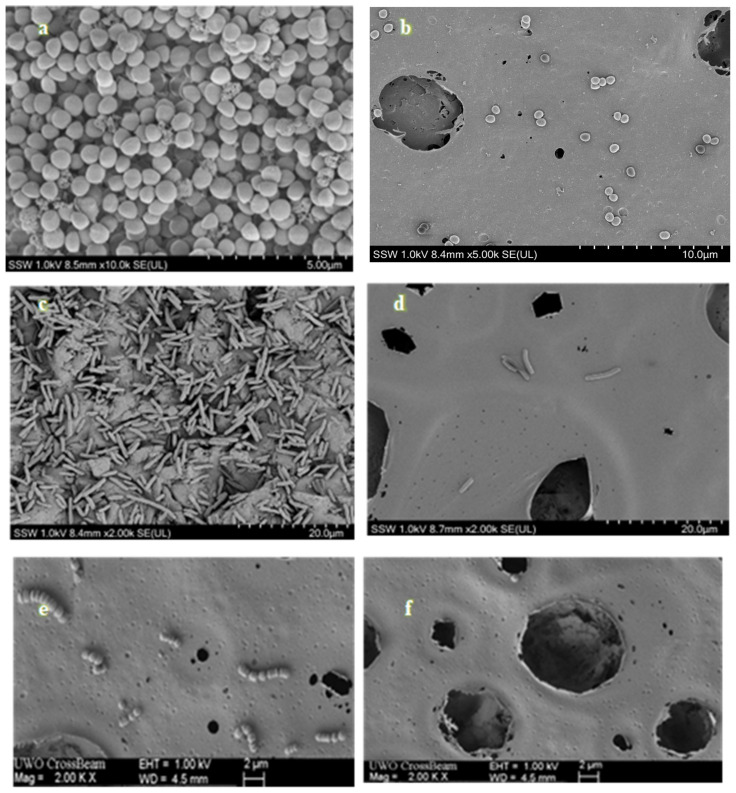
SEM images of biofilms formed on MMA surface treated with (**a**) *S. aureus*, (**c**) *E. coli.*, and (**e**) *S. agalactiae*, respectively, after 24 h. SEM images of biofilms on MMA/CBD disc surface treated with (**b**) *S. aureus*, (**d**) *E. coli*, and (**f**) *S. agalactiae* after 24 h.

The results of the bacterial biofilm formation experiments are presented in Figure 3. The largest significant difference in bacterial adhesion and growth between the CBD/PMMA-coated disc and PMMA-coated disc was on *S. agalactiae* biofilms. *S. agalactiae* growth on the PMMA surface was significantly 10,000 times greater compared with the PMMA/CBD discs. That is why we rarely see any bacteria on the surface of *S. agalactiae* in Figure 4. This was confirmed with an unpaired *t*-test with *p* < 0.0001. CBD-enriched coatings in the PMMA reduced *S. agalactiae* biofilms to 99.99%. 

**Quantitative Evaluation of Inhibition in Gram-Positive Biofilms:** In the *S. aureus* group, their biofilm was reduced 100 times (2 log fold) on PMMA/CBD surfaces as compared to PMMA surfaces, which is why we see some bacteria in Figure 4a as compared to Figure 4d. *t*-tests where *p* < 0.0001 was used to confirm the results. The addition of CBD in the coatings reduced *S. aureus* biofilms by 99%.

Overall, *S. agalactiae* had the lowest growth on the synthesized surfaces, and bacteria were observed to be formed less on the media surrounding the surface. *E. coli* showed the least biofilm attached to the surface, although there was significant growth on the media surrounding the disc. The most significant difference between materials was found in *S. agalactiae* with a reduction of 99.99% biofilm formation. The results match the SEM images in Section 2.4.2 where there are no *S. agalactiae* biofilms in the CBD/PMMA coatings, although we noticed some growth in the PMMA coatings. Equivalent levels of bacterial proliferation were observed in both *S. aureus* and *E. coli* in both the scanning electron microscopy (SEM) visuals and the quantitative assessments of the CBD/PMMA coatings.

**Quantitative Evaluation of Inhibition in Gram-Negative Biofilms**: As for the Gram-negative bacteria, *E. coli* biofilms had reduced attachment on the PMMA/CBD surface, but there remained significant amounts of biofilm attached to the media surrounding the surface. *E. coli* biofilms were reduced 100 times (2 log fold) on PMMA/CBD surfaces as compared to PMMA surfaces. Unpaired *t*-tests with *p* < 0.0001 were used to confirm the results that the addition of CBD in PMMA significantly reduced *E. coli* biofilm amounts. The addition of CBD in the coatings reduced *E. coli* biofilms from growing and attaching by 99%. Table 2 shows the percentage reduction profiles of PMMA/CBD surfaces for all bacterial strains used in this article.

#### 2.4.2. Visual Analysis of Biofilms on Coating

The SEM images of Figure 4 depict a representative field of view at magnifications of 10,000× and 2000×, respectively, for each surface treated with *S. agalactiae*, *S. aureus*, and *E. coli*. Additionally, within the same figure, we showcase selected representative fields of view for the biofilm formed on both PMMA-coated (control) and PMMA/CBD-coated discs after 24 h of incubation. The contrast in the quantity of biofilm volume between the only PMMA-coated and the PMMA/CBD-coated surfaces is readily apparent and aligns with the quantitative data in Section 2.4.1. On surfaces coated with PMMA only, microbial cells were organized in mono- and multi-layered structures, covering most of the surface area, regardless of the type of strain (Figure 4a,c,e). Conversely, in CBD/PMMA-coated samples, there was rarely a presence of bacterial cells, either singular or forming small clusters on the surface. For surfaces treated with *S. agalactiae*, the bacteria were primarily concentrated within the crevices and recesses of the coating (illustrated in Figure 4f). The least number of microbial cells on the surface was observed on the CBD/PMMA disc surface when incubated with the *E. coli* strain (Figure 4d). 

***Overview of CBD’s Antibiofilm Effects on Gram-Negative Biofilms****:* Biofilms are usually more resistant (resistant (100–1000-fold)) to drugs compared to their planktonic counterparts. Biofilm formation in *E. coli* is a complex developmental process that occurs in different phases: reversible and irreversible attachment, maturation, and dispersion [32]. There were no *E. coli* biofilms observed on the PMMA/CBD coatings, although there was a significant amount of biofilm observed on the media surrounding the disc. This was due to the CBD in the coating inhibiting the adhesion of the planktonic bacteria, which is the initial stage of biofilm formation. This is an excellent preventive strategy to control biofilms. CBD has been reported to affect gene expressions involved in biofilm maintenance, development, and maturation of factors associated with exopolysaccharide (EPS) synthesis [33]. EPSs establish the functional and structural integrity of biofilms and are considered the fundamental component that determines the physicochemical properties of a biofilm. CBD may modify the architecture of *E. coli* biofilms as well, by reducing its thickness and exopolysaccharide (EPS) production accompanied by downregulation of genes involved in EPS synthesis. CBD may affect the biosynthesis of fimbriae, surface proteins, virulence factor genes, and other bacterial structures involved in adherence to surfaces [31]. CBD does not inhibit planktonic *E. coli* cells as it targets the genes required for synthesizing fimbriae and EPSs [34]. Fimbriae are not necessary for planktonic bacteria as planktonic bacteria do not typically require attachment to surfaces or host cells. Planktonic bacteria exist as free-floating individual cells in a liquid medium, where they can move and disperse freely. Both fimbriae and EPS production primarily facilitate adhesion and attachment to surfaces, host tissues, or other cells, which is essential for processes such as biofilm formation and infection. Since planktonic bacteria do not need to adhere to surfaces or host tissues, they do not require these features for their survival or growth in the planktonic state. CBD may target these genes and be effective only for biofilms; it still remains ineffective against planktonic bacteria as they may still possess fimbriae or similar structures, albeit in reduced or modified forms. These structures might serve alternative functions in the planktonic lifestyle, such as facilitating interactions with other bacteria or sensing environmental cues. Nevertheless, since these structures are not typically considered necessary for the planktonic mode of growth, CBD remains ineffective for *E. coli* in the planktonic phase as shown in Section 2.3 and Section 2.4.

***Overview of Evaluation of CBD’s Antibiofilm Effects on Gram-Positive Biofilms****:* Gram-positive bacteria’s bacterial membrane properties, including hydrophobicity, are crucial for initial bacterial adhesion, a critical step in biofilm development. CBD/PMMA coatings have shown to be very hydrophobic and could target bacterial cell-surface hydrophobicity. Recent studies [35] showed that endocannabinoids effectively modified the cell-surface properties of MRSA strains, reducing their hydrophobicity, which contributed to antibiofilm activity, as higher cell-surface hydrophobicity has been linked to increased biofilm formation levels. Endocannabinoids and CBD are both cannabinoids, but the production is in different hosts—CBD is plant sourced, whereas endocannabinoids are endogenous [36]. Similarly, CBD must also modify the cell membrane properties of *S. agalactiae* and *S. aureus*, which reduces their potential to form biofilms. It was already stated in Section 2.4 that CBD changes the membrane potential, causing membrane depolarization. In this study, we propose that the mechanisms of action against planktonic bacteria of the coatings we tested are due to their ability to modify the bacterial membrane potential, which then leads to changes in biofilm-related characteristics of Gram-positive bacteria, such as hydrophobicity and cell aggregation.

### 2.5. CBD Release Profiles

**Figure 5:**
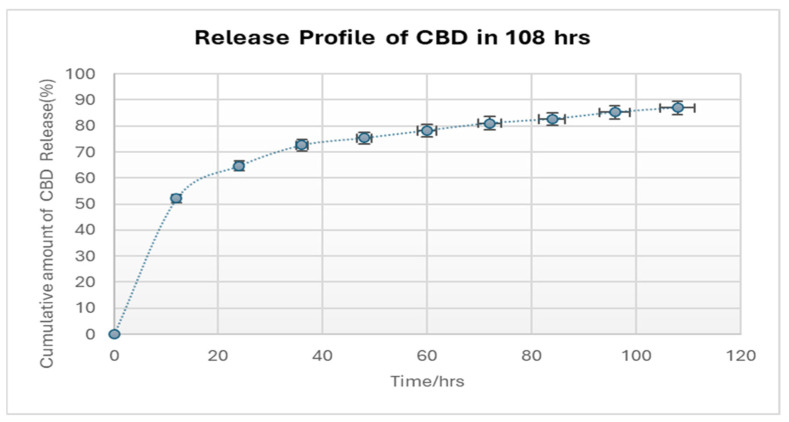
Release profile of 1000 mg of CBD from CBD/PMMA coating in 100 mL water solution (% error: 5%).

The in vitro CBD release characteristics of the CBD/PMMA coatings were studied. Dissolution data for all the experiments were highly reproducible, and hence, only the average values were plotted. The synthesized coatings were examined for release of CBD as shown in Figure 5. The three coated discs of CBD/PMMA were shown to have nearly identical cumulative release profiles; after a high initial release of CBD, a reduced yet constant sustained release was observed. In the first 12 h, 50% of the CBD was released promptly for 1000 mg loaded CBD coating, which could be attributed to the dissolution of the adsorbed particles or to the diffusion of CBD located close to the surface of the coating. Since the coating was thin (<0.5 mm), it is probable that most of the CBD molecules were entrapped close to the surface of the coating. This factor could facilitate the observed initial burst release [37]. The burst phase lasted ≈12 h. Another 50–80.5% was released in a slow linear-like fashion over the rest of the 2 days, and after 80 h of CBD release, the release rate became constant. In our system, the CBD released from the coating lasted for almost 108 h. Hence, the coating was found to have the ability to deliver antimicrobial agents in a controlled manner. The observed burst effect is the reason behind the antibiofilm capacity of the coating. The interaction between antibiofilm agents and the burst effect of drugs is a critical consideration in combating bacterial infections, particularly those associated with biofilm formation. In the case of antibiofilm CBD, a burst release could potentially lead to a rapid disruption of the biofilm matrix, allowing bacteria to be quickly eradicated as shown in Figure 4.

## 3. Materials and Methods

### 3.1. Chemicals, Culture Media, and Microorganisms

Cannabidiol (CBD) isolate was supplied by the Mera Cannabis Corp, Toronto, ON, Canada. Methyl methacrylate (MMA, 99%) and photo initiator, 2-hydroxy-2-methylpropiophenone (HMP, 97%), poly(methyl methacrylate) (PMMA, Mw = 350,000 Da), Tryptic Soy Agar (TSA), Tryptic Soy Broth, and LB miller’s formulation culture medium were purchased from Sigma-Aldrich, Oakville, ON, Canada. Acetonitrile and chloroform were purchased from Caledon Laboratory Ltd., Georgetown, ON, Canada. Glucose Anhydrous (powder) was purchased from Thermo Fisher Scientific (Whitby, ON, Canada) and was dissolved in deionized water. Pathogenic *Staphylococcus aureus* (*S. aureus*) (ATCC^®^ 29213™), *Escherichia coli* (*E. coli*) (ATCC^®^ 25922™), and *Streptococcus agalactiae* (*S. agalactiae*) (ATCC^®^ 29213™) were used from Heinrichs Lab stock (Microbiology and Immunology, Western University, London, ON, Canada).

### 3.2. Isolation and Characterization of CBD

CBD samples were purified and characterized as outlined in [38,39]. To assess the purity of the obtained CBD crystal samples, samples were conducted using ^1^H NMR spectroscopy on a Varian Inova—600 (Agilent, Santa Clara, CA, USA).

### 3.3. Synthesis of MMA and CBD Coatings

An amount of 0.62 g of CBD was dissolved in 1.001 g of MMA, with a molar ratio of 1:0.2. Then, 0.08 g of PMMA and 0.0328 g of photo initiator HMP were added. PMMA was used as a thickener to increase the viscosity of the reaction mixture. Then, 0.2 mL of the mixture was dropped and evenly spread onto a 15 mm in diameter and 1 mm in thickness titanium disc, pre-cleaned by sonication in ethanol for 20 min and air-dried. The use of metal discs was to facilitate SEM imaging feasibility. The thin layer of coating on the titanium disc was cured inside a flat-bottom Teflon evaporating dish covered with a quartz lid under UV irradiation (UVP 95-0127-01 Blak-Ray™ B- 100AP lamp, 100 W, 365 nm longwave, UVP, LLC, Upland, CA, USA) for 1 min, 5 min, and 15 min. The evaporating dish was purged with nitrogen gas before and during UV curing by means of a plastic tube. After that, the coated disc was placed in a vacuum oven to remove any remaining VOCs in the coating. In the preparation of all coating samples, the intensity of the UV light received by the coating was measured to be 8335 μW/cm^2^ by a Sper Scientific 850,009 UVA/B light meter (Tempe, AZ, USA). The distance from the UV light source to the coating surface was 4 cm.

### 3.4. Coating Characterization

Nuclear magnetic resonance (NMR) spectra were collected using a Bruker Avance III HD 400 spectrometer (Billerica, MA, USA). CBD/PMMA coatings were dissolved in deuterated chloroform. The morphologies and surfaces of the coating particles were tested by scanning electron microscopy (SEM) and analyzed by ImageJ Software, Java 1.8. 64-bit, to determine the best ratio of CBD/PMMA for forming uniform coating. SEM images of CBD/PMMA coatings were taken before/after exposure to fluids to examine the surface morphology following exposure to fluids, assessing whether the leaching of the CBD component occurred for enhanced biofilm efficacy. CBD leaching is necessary for the coating to function in combating pathogenic biofilms.

### 3.5. Antimicrobial Activity of CBD

#### 3.5.1. Zone of Inhibition Studies

This approach was used to validate the antimicrobial potential of CBD on the tested pathogens. The agar disk diffusion method [40] was used to evaluate cannabidiol solutions in DMSO. Agar plates were inoculated with a standardized O.D 1 inoculum of *S. aureus*, *E. coli*, and *S. agalactiae*. Filter paper discs (6 mm dia.) containing 10 µL CBD solution in DMSO at a concentration of 20 mg/mL and 200 mg/mL (amount like the CBD release studies in Section 3.8 were placed on the agar surface. The petri dishes were incubated overnight in 37 °C. The antimicrobial CBD diffused into the agar media and inhibited the growth of the microorganism which was measured by the diameter of the inhibition growth zone and then recorded. The solvent, DMSO, was used as the control for inhibition zone studies as well.

#### 3.5.2. Modified Minimum Inhibitory Concentration (MIC)

This approach was used to assess the efficiency of CBD against pathogenic *S. aureus*, *E. coli*, and *S. agalactiae* before using it for coating purposes. To determine the MIC for CBD, we dissolved it in warm DMSO to make concentrations of 20, 10, 5, 2.5, 1, and 0.6 µg/mL. The various concentrations of CBD in DMSO solution were added to 1 mL of Tryptic soy broth (TSB) growth media. DMSO was used as control. Then, 10 µL of each bacteria at an OD_600_ 1 was added to each sample, reaching a starting OD_600_ of 0.1. The samples were then incubated at 37 °C with shaking for 24 h. The OD of the samples were then measured in a 96-well plate using a microplate spectrophotometer from Biotek instruments (Winooski, VT, USA), and the MIC was determined as the lowest concentration at which no discernible growth was recorded. The experiment was repeated in three separate trials and the results were recorded.

### 3.6. Bactericidal Activity of CBD

Minimum bactericidal concentration for CBD was also determined by preparing solutions in DMSO similar to the procedure described in Section 3.5. Prepared CBD samples with concentrations ranging from 20, 10, 5, 2.5, to 1 mg/mL were tested against pathogenic *S. aureus* (ATCC^®^ 29213™), *E. coli* (ATCC^®^ 25922™), and *S. agalactiae* (ATCC^®^ 10231™), which were added to each sample at an initial OD_600_ 0.01. The samples underwent incubation at 37 °C for 24 h. After incubation, the samples were plated and their respective colonies were counted and recorded. The MBC was defined as the lowest concentration of CBD that eradicated 99.9% of the original inoculum with no colony forming unit being observed when plated. The experiment was replicated in three distinct trials, and the outcomes were graphed using GraphPad Prism (San Diego, CA, USA).

### 3.7. Biofilm Studies of PMMA/CBD Coatings

Three strains, *S. aureus* (ATCC^®^ 29213™), *E*. *coli* (ATCC^®^ 25922™), and *S. agalactiae* (ATCC^®^ 10231™), were used in this study, given their ability of producing high amounts of slime according to the methods and criteria proposed in [41]. Strains were stored at −80 °C (Heinrichs Lab, Western University) and grown on Tryptic Soy Agar (TSA) plates at 37 °C for 20 h before experimental assay.

*S. aureus* and *S. agalactiae* suspensions at the concentration of 1 × 10^7^ Colony Forming Unit per mL (CFU/mL) were prepared in TSB supplemented with 40% *w*/*v* glucose (Fisher Chemicals, Waltham, MA, USA), while L.B. was used for *E. coli* experiments, to induce slime production [41].

**Figure 6:**
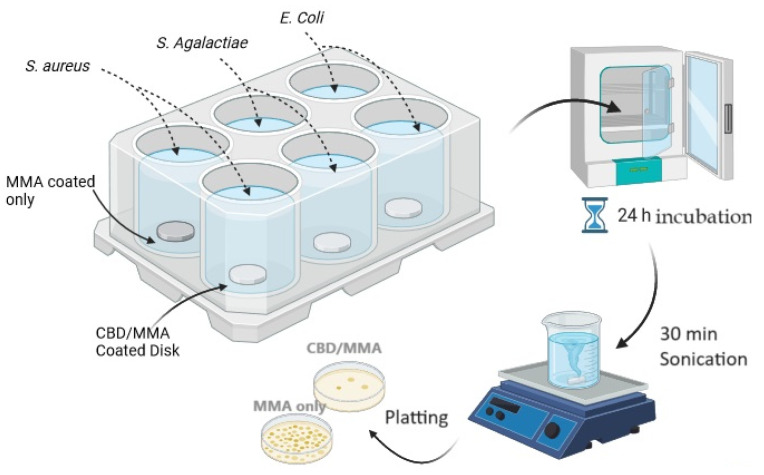
Diagram of the protocol used to test synthesized coatings on biofilms of *S. aureus*, *E. coli*, and *S. agalactiae*.

Biofilm formation on the surface of the coated discs (both PMMA/CBD-coated and PMMA-coated control discs) was obtained in 6-well plates, fitting one disc for each well. Then, 20 µL of bacterial suspension, 10 µL of a 40% (*v*/*v*) glucose solution, and 2 mL broth media were added to each well, thus guaranteeing submersion of the disc into the medium with bacteria. Plates were then immediately incubated at 37 °C for 24 h under static conditions. Two independent replicate experiments were conducted. Figure 6 shows the workflow for the biofilm studies.

#### 3.7.1. Visualization of Biofilm Formation by Scanning Electron Microscopy

Biofilm formation on the coated and uncoated surfaces after 24 h of incubation was imaged with scanning electron microscopy (SEM) for visual assessment. After incubation, the samples were fixed in 2% paraformaldehyde and 2.8% glutaraldehyde for 1 h at room temperature followed by a washing step with 0.1 M HEPES buffer 3 times and slow dehydration using 30%, 50%, 70%, 95%, and 100% ethanol in series. In order to reduce sample surface tension, samples were immersed in 25%, 50%, 75%, 90%, and 100% hexamethyldisilane (Sigma-Aldrich) for 15 min and evaporated overnight under the fume hood, before mounting on aluminum SEM stubs and sputter-coating with a 10 nm gold–palladium layer, using a Leica EM ACE600 sputter coater (Leica Microsystems, Wetzlar, Germany). Pictures of experiments with *Staphylococcus aureus* and *Escherichia coli* were taken at random locations with various magnifications depending on the bacterial size using scanning electron microscopy coupled with energy dispersive X-ray spectroscopy (SEM/EDX) using a Hitachi (Tokyo, Japan) SU8230 Regulus ultra-high resolution field emission scanning electron microscope (FESEM) combined with a Bruker X-Flash 6160 SSD X-ray detector. Pictures of experiments with *S. agalactiae* were taken with a Zeiss 1540XB Cross Beam (Jena, Germany).

#### 3.7.2. Quantitative Tests for Biofilm Formation

After the 6-well plates were incubated and the biofilms were formed, the samples were gently tilted to remove nonadherent bacteria. For quantitative culture, bacteria were subsequently detached from the coated discs in a sterile tube with 4 mL of PBS by 30 min of sonication (Elma Transonic T460, 35 kHz; Elma Schmidbauer GmbH, Singen, Germany). This detachment method was highly efficient, resulted in the removal of the vast majority of bacteria attached, and seemed comparable between PMMA/CBD and MMA. Ten-fold serial dilutions of each sonicated solution were made in PBS and triplicate 10 μL aliquots of the sample and the dilutions were spotted on TSA agar plates. The plates were incubated overnight at 37 °C and the number of CFU/implant were determined and the tables were plotted.

### 3.8. CBD Release Studies

#### 3.8.1. Standard Solutions and Calibration Curves

The protocol mentioned in [42] was followed to study the drug release of CBD from the coating. Different working standards, namely, 0.033 mg/mL, 0.10 mg/mL, 0.2 mg/mL, 0.4 mg/mL, and 0.6 mg/mL, were prepared by appropriate dilutions. Absorbance of those solutions at the λ_max_ 205 nm was measured, which is close to the λ_max_ for CBD stated in [43].

For the calibration curve, accurately weighed 3 mg of CBD was transferred to a vial of 1 mL deionized water solution. From this solution, other solutions of different concentrations in mg/mL were obtained by diluting adequate amounts in triplicate. Five point’s calibration graphs were constructed covering a concentration range of 33–600 µg/mL. Three independent determinations were performed at each concentration. Linear relationships between the absorbance and the corresponding drug concentration were observed and the absorbance–concentration equation was noted for determining the coefficient (r^2^).

Linear relationships between the absorbance and the corresponding CBD concentration were observed. It is important to highlight that CBD–absorbance relationships do not adhere to linear patterns when concentrations surpass 800 µg/mL. This means that increasing the dosage beyond this threshold makes it very difficult to dissolve in water-based solvents due to its fat-soluble nature. The standard deviations of the slope and intercept were low. The determination coefficient (r2) was 0.997 (Figure S2 Supplementary Information).

#### 3.8.2. In Vitro Release Studies

The in vitro dissolution studies of the CBD/PMMA coatings were carried out using protocol mentioned in [42]. The dissolution medium consisted of 100 mL of deionized water maintained at 25 ± 0.5 °C. The CBD released at different time intervals was measured using a UV–visible spectrophotometer (Shimadzu UV–Vis 3600, Washington, DC, USA). It was made clear that none of the coating compounds (PMMA, unreacted MMA) used in the coating formulations interfered with the absorbance of the CBD in water. The dissolution jars were cleaned with acetone and then rinsed with distilled water and dried to room temperature. Then, 100 mL of dissolution medium (deionized water) was transferred into the dissolution jars and the coated discs were placed in the jars and a static position was maintained. An amount of 3 mL of each sample was withdrawn at 12 h intervals such as 0, 12, 24, 36, 48, 60, 72, 84, 96, and 108 h using a graduated pipette and transferred to clean, dried, and labeled test tubes. All the samples were then measured at 205 nm and their corresponding absorbance was noted. The cumulative percentage of released CBD was calculated using the given formulas as stated in [42]:

Concentration of CBD released (mg/mL)=(slope×Absorbance)±intercept

$$Cumulative\;Percentage\;release\left(\%\right)=\frac{Volume\;of\;sample\;withdrawn\;(mL)}{Bath\;volume\;(mL)}\times P\left(t\right)+P(t-1)$$

$$P\left(t\right)=percentage\;release\;at\;time\;t$$

where, t=0,12,24,36,48,60,72,84,96, and 108 h,

$$P\left(t-1\right)=percentage\;release\;previous\;to\;time\;t.$$

### 3.9. Statistical Analysis

All statistical analyses were performed with Prism Software version 8.3. The mean number of CFUs recultured from discs of PMMA and PMMA/CBD were compared by unpaired *t*-test. Comparisons between the control and solvent column with the different concentrations of CBD were also conducted using ANOVA multiple analysis (Tukey’s model). There was a significant difference between the values with CBD and without CBD, and the *p*-values were shown in the graphs. A *p*-value of <0.05 was statistically significant. Graphs were created with GraphPad Prism 8.3.0 (GraphPad Software, La Jolla, CA, USA).

## 4. Conclusions

This investigation presents a pioneering approach for the development of antibiotic-free coatings, offering an alternative to conventional methods. Although attempts to copolymerize CBD with MMA have been ineffective, CBD was physically incorporated into PMMA coatings using UV curing. Analysis via scanning electron microscopy (SEM) indicated that, in addition to CBD concentration, reaction duration potentially influences the smoothness of the coating surface. Future research could be explored on the impact of curing time in CBD/PMMA synthesis, surface texture, and regular distribution.

Emphasis should be placed on crafting biofilm coatings capable of sustained release of antibiofilm agents. This sustained release will maintain effective concentrations of the CBD over an extended period, maximizing their ability to disrupt biofilms and eliminate bacteria within them. Future strategies to mitigate the burst effect could be optimizing formulation parameters such as adding modifiers, surfactants, or stabilizers to the formulation that can influence the release kinetics by altering the solubility, diffusion, or interactions of CBD within the formulation. Implementing controlled release technologies such as microencapsulation, nanoparticles, liposomes, or hydrogels could be used to regulate the release of the CBD over time. These technologies can provide a barrier to fast diffusion or degradation, resulting in a sustained and controlled release profile. By carefully designing the delivery system, it is possible to minimize the initial burst release and achieve more predictable drug release kinetics. Further studies can be performed to analyze coating techniques such as supercritical impregnation of CBD in PMMA for uniformity and durability of the coating [44].

In summary, biofilm studies showed PMMA/CBD coatings were effective in eradicating all the pathogens on their surface. Antimicrobial studies showed CBD could not inhibit planktonic *E. coli* cells but was effective against *E. coli* biofilms. Further investigations on the gene expressions involved in biofilm maintenance, development, and maturation of factors associated with exopolysaccharide (EPS) synthesis and CBD could be studied to better understand its antibiofilm mechanisms. Other scopes of research could be CBD and its effect on the biosynthesis of fimbriae, surface proteins, virulence factor genes, and other bacterial structures involved in adherence to surfaces.

This study shows a novel pathway to develop an alternative to antibiotic-filled coatings. CBD was successfully incorporated into the PMMA coatings using UV radiation and a photo initiator under N_2_. CBD-loaded PMMA coatings showed strong antibiofilm activities for both Gram-negative as well as Gram-positive bacteria, as confirmed by SEM and quantitative analyses of the surfaces. The addition of CBD in the coatings provided a 99% reduction in *S. aureus* and *E. coli* biofilms, and a 99.99% reduction in *S. agalactiae* biofilms. The CBD release analysis revealed a burst release for the first 12 h and then a steady release into the surrounding environment in vitro afterward. The efficacy of such antimicrobial medicines in coatings is dependent on the antibiotic dissolving in fluids before absorption into the systemic circulation. The rate of CBD dissolution in the coating was consequently critical. In vitro, the data reveal that CBD molecules are released from the coatings quite effectively. This study showed that CBD can be incorporated into materials, enabling antibiotic-free dentures for preventing dental biofilms and reducing dental plaques.

## Figures and Tables

**Table 1:** Minimum inhibitory conc/minimum bactericidal conc. of cannabidiol and inhibition zones of cannabidiol.

Strain	MIC (µg/mL)	MBC (µg/mL)	ZOI (mm) at 20 mg	ZOI (mm) at 200 mg
*S. aureus*	2.5 ± 0.4	20 ± 0.5	11 ± 1 mm	20 ± 1
*S. agalactiae*	2 ± 0.1	10 ± 0.4	9 ± 1 mm	17 ± 2
*E. coli*	Resistant	Resistant	No Zones	No zones

All the treatments were performed in triplicate. Means and standard deviations are reported as mean ± SD.

**Table 2:** Percentage growth inhibition of *S. aureus*, *S. agalactiae*, and *E. coli* on PMMA/CBD surfaces.

Strain	% Reduction of Biofilm on PMMA/CBD Coating
*S. aureus*	99%
*S. agalactiae*	99.99%
*E. coli*	99%

## Data Availability

Data are contained within the article and Supplementary Materials.

## Supplementary Materials

The following supporting information can be downloaded at: https://www.mdpi.com/article/10.3390/molecules30040943/s1, Figure S1. ^1^H NMR spectrum of CBD in Chloroform-d. Figure S2. Calibration graph of CBD Concentration versus Absorbance. Figure S3. Experimental setup for UV curing of coatings.
