# Supplementary material for: Antimicrobial Denture Material Synthesized from Poly(methyl methacrylate) Enriched with Cannabidiol Isolates
Source: Molecules. 2025 Feb 18;30(4):943. doi: 10.3390/molecules30040943 (PMC11858198; doi:10.3390/molecules30040943)

Supplementary Materials for Article

Antimicrobial Denture Material Synthesized from Poly(Methyl Methacrylate) Enriched with Cannabidiol Isolates

Kazi Tahsin [1,*], William Xu [2], David Watson [3], Amin Rizkalla [1,2] and Paul Charpentier [1,2,*]

[1] Biomedical Engineering, Western University, London, ON N6A 3K7, Canada; arizkall@uwo.ca
[2] Chemical & Biochemical Engineering, Western University, London, ON N6A 5B9, Canada; zxu27@uwo.ca
[3] Microbiology & Immunology, Western University, London, ON N6A 5C1, Canada; dwatso25@uwo.ca
* Correspondence: ktahsin@uwo.ca (K.T.); pcharpen@uwo.ca (P.C.); Tel.: +1-519-661-2111 (ext. 83466) (P.C.)

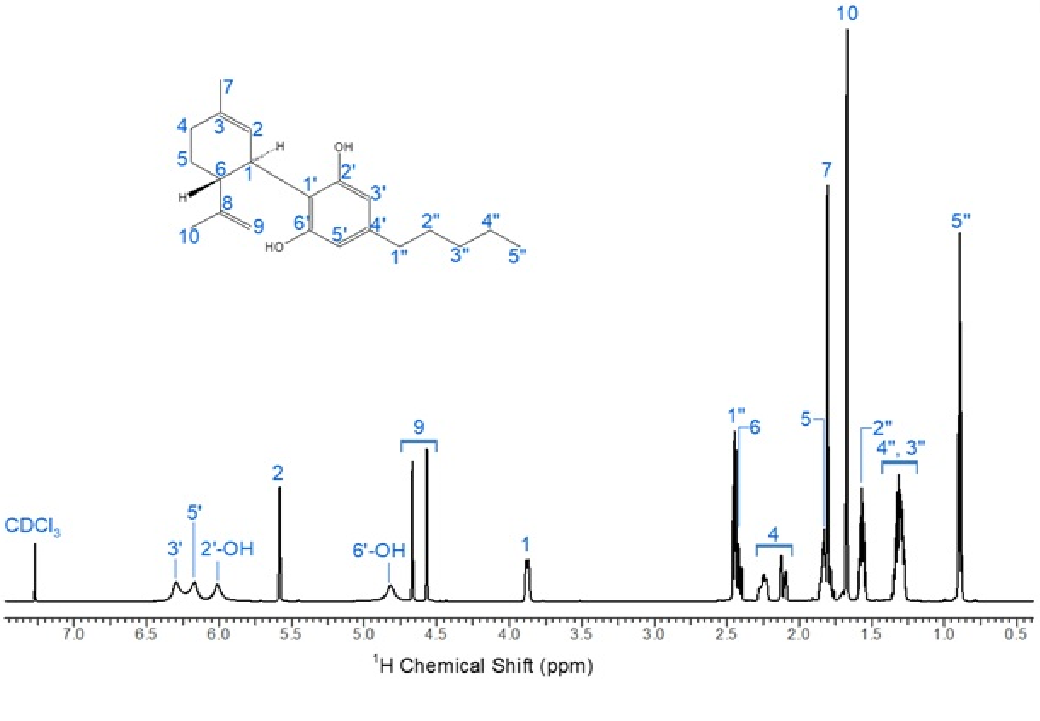

Figure S1. ^{1}H NMR spectrum of CBD in Chloroform - d

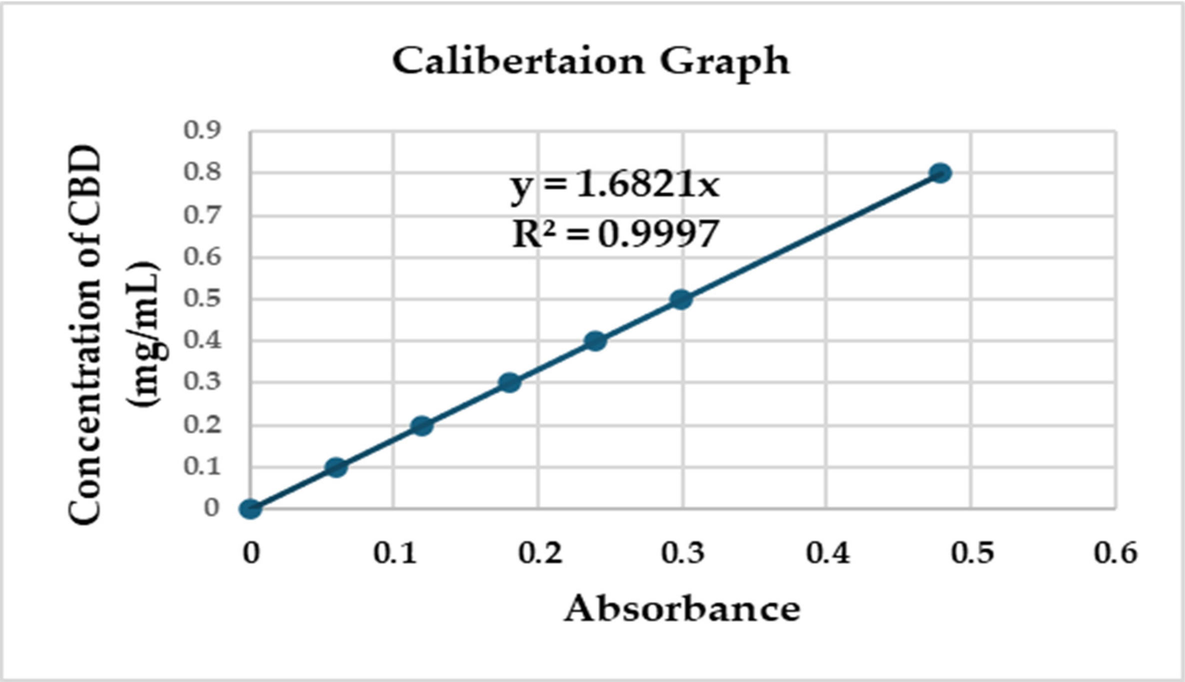

Figure S2. Calibration graph of CBD Concentration versus Absorbance

Figure S3. Experimental setup for UV curing of coatings.

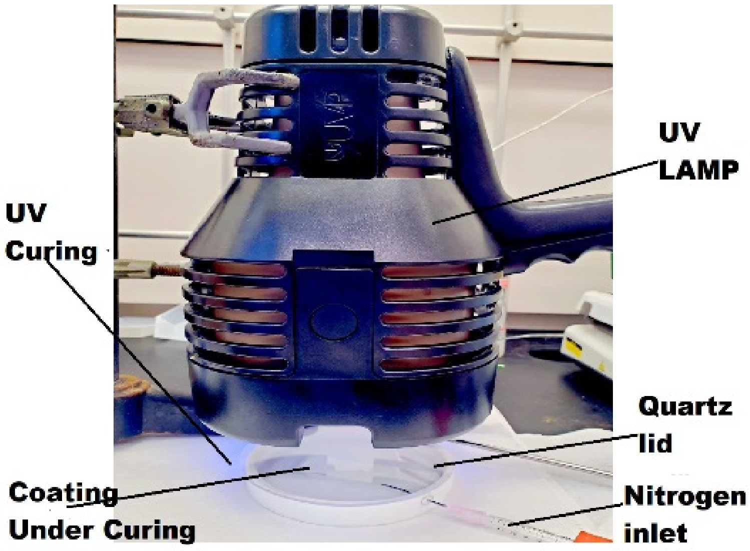

Supplement: Supplementary file 1 [file molecules-30-00943-s001.zip › molecules-3396941-supplementary.pdf]
